# Experimental and numerical dataset of Microbond test using optical fibres for strain

**DOI:** 10.1016/j.dib.2020.106017

**Published:** 2020-07-13

**Authors:** R. Dsouza, P. Antunes, M. Kakkonen, J. Jokinen, E. Sarlin, P. Kallio, M. Kanerva

**Affiliations:** a Tampere University, Faculty of Engineering and Natural Sciences, P.O.Box 589, FI-33014 Tampere, Finland; b Fibrobotics Oy, Korkeakoulunkatu 1, 33720 Tampere, Finland; c Tampere University, Faculty of Medicine and Health Technology, P.O.Box 589, FI-33014 Tampere, Finland; d Instituto de Telecomunicações - Aveiro, PO Box 3810-193, Aveiro, Portugal; e Physics Department and I3N, Aveiro University, Campus de Santiago, PO Box 3810-193, Aveiro, Portugal

**Keywords:** Optical fibres, Finite element analysis (FEA), Cohesive Zone Modelling, Debonding, Interface

## Abstract

This data article provides useful information often required for numerical modeling of the so-called microbond tests. It includes the experimental and simulation data of the microbond testing using Fibre Bragg Grating (FBG) fibres for optical strains. Microbond testing was performed on five different droplets of varying embedded length and diameter to collect the data. Finite element simulation was carried out and modelling was validated, by using two variables force and strain, to collect the data. The output data of the fitted models is given and is also visualized via graphs of force-strain derivative curves. The data of the simulations is provided for different finite element mesh densities. Here, to clarify the type and form of the data for the use by readers, the energy distribution curves describing various functionalities of the droplet, fibre and interface are presented. For further reading, the interpretation and analysis of this data can be found in a research article titled “3D interfacial debonding during microbond testing: Advantages of local strain recording” (R. Dsouza et al., 2020) [1].

Specifications Table

**Table:** 

Subject area	Modelling and Simulation
Specific subject area	Interface failure analysis
Type of data	Table Image Chart Graph Figure Output data files
How data was acquired	Mechanical testing, finite element analysis (Abaqus, Standard, version 2017), optical camera (model UI-3370SE, IDS, Germany), strain acquisition system (W3/1050 Series Fiber Bragg Grating Interrogator, Smart Fibers^Ⓡ^) Scanning Electron Microscopy (model ULTRAplus, Zeiss, Germany)
Data format	Raw Analyzed Filtered Visualizations
Parameters for data collection	Experimental parameters and finite element method related parameters
Description of data collection	Experimental and numerical data collection and exported output data from microbond tests
Data source location	City: Tampere Country: Finland
Data accessibility	With the article
Related research article	Dsouza, R, Antunes, P., Kakkonen, M., Jokinen, J., Sarlin, E., Kallio, P. and Kanerva, M. 3D interfacial debonding during microbond testing: advantages of local strain recording, Journal of Composite Science and Technology, Volume 195, 108163 (2020).

## Value of the data

• The data were generated using complex and computationally expensive numerical methods and can be of use to researchers that are interested in understanding the 3D microbond test.
• Simulated force-strain data for different droplets allows one to understand the behavior of the models.
• Finite element (FE) analysis with high mesh density can be useful for researchers to understand the effect of mesh size in the microbond FE model.
• Microscopy images and FE simulations of droplets give valuable information on the effectiveness of the material parameters used in FE models.

## Data

The data of this work includes multiple sets of simulated and experimental data. A detailed description of the data is given in Table 1**.** The following sub chapters include the representations of the data (described in Table 1) to indicate the type and relations in the data (e.g. experimental tests and indications of corresponding simulations). The details of the experimental methods and modelling inputs (numerical parameters) of finite element analysis (FEA) to collect the data are given in Chapter 2 about the method details.

**Table 1:** Description of data and visualizations of this dataset.

Context	Page no.	Re-presentation	Data files
Experimental force-strain data	4	Force-strain curves	Experimental_data.xlsx
Experimental load-embedded area	5	Debond load-embedded area data points	Load_area.txt
Estimation of fracture energy	6	Tabular data	-
FEA of force-strain data	6	Force-displacement and strain-displacement data	force_strain_sim.xlsx, simulation_data.xlsx
Different blade position data	7	Force-displacement and strain-displacement data	DR_3_position1.mp4, DR_3_position2.mp4, DR_3_position3.mp4
Mesh density data	8	Force-strain curves	fine_mesh.xlsx, microbond_1.mp4
Microscopy data	8	Scanning electron microscope (SEM) images	SEM_1.tif, SEM_2.tif, SEM_4.tif, SEM_5.tif
Force-strain derivative data	11	Graphical data	-

## Experimental design, materials, and methods

**Fig. 1:**
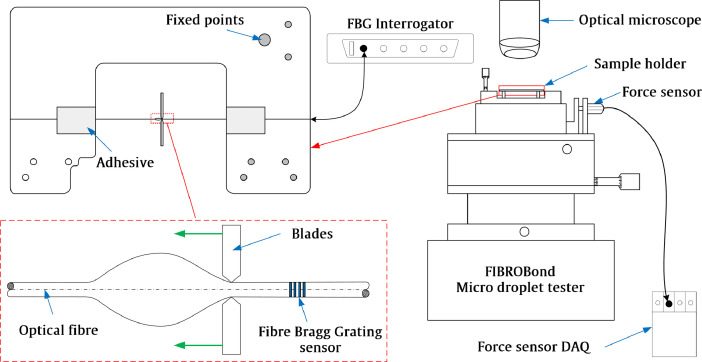
Experimental setup of the MB test to collect data about the interface.

Fibre matrix interfaces form a crucial part of composite material, as the interfacial adhesion is an ongoing investigation from the past three decades. Interfacial adhesion affects the laminate level performance of the composite. One such micromechanical test is the Microbond test (MB), which is widely used [2] and in focus here. The traditional MB test consists of only one output, ‘Force’, whereas the current work has established the usage and functionality of strain. Strain makes the test having two output parameters. Here, the experimental setup consisted of Fibre Bragg Grating (FBG) optical fibre embedded with five droplets of varying geometry. The schematic of the experimental setup is described in Fig. 1. The droplets were made of Araldite^Ⓡ^ LY5052, as resin, and Aradur^Ⓡ^5052, as hardener. MB test was carried on in the FIBRObond microdroplet tester. The force data was recorded using the FIBRObond microdroplet tester [2], which has been developed by Fibrobotics (Tampere, Finland). The strain data was recorded using a W3/1050 series Fiber Bragg Grating Interrogator (Smart Fibers^Ⓡ^) with a remote interface W3 WDM (version 1.04). The force and strain data was recorded at a sampling rate of 50 Hz. The exact details are presented in the accompanying article [1]. SEM was carried out on droplets after the experiments. Prior to SEM studies, the specimens were coated with a thin layer of carbon to avoid charging.

**Fig. 2:**
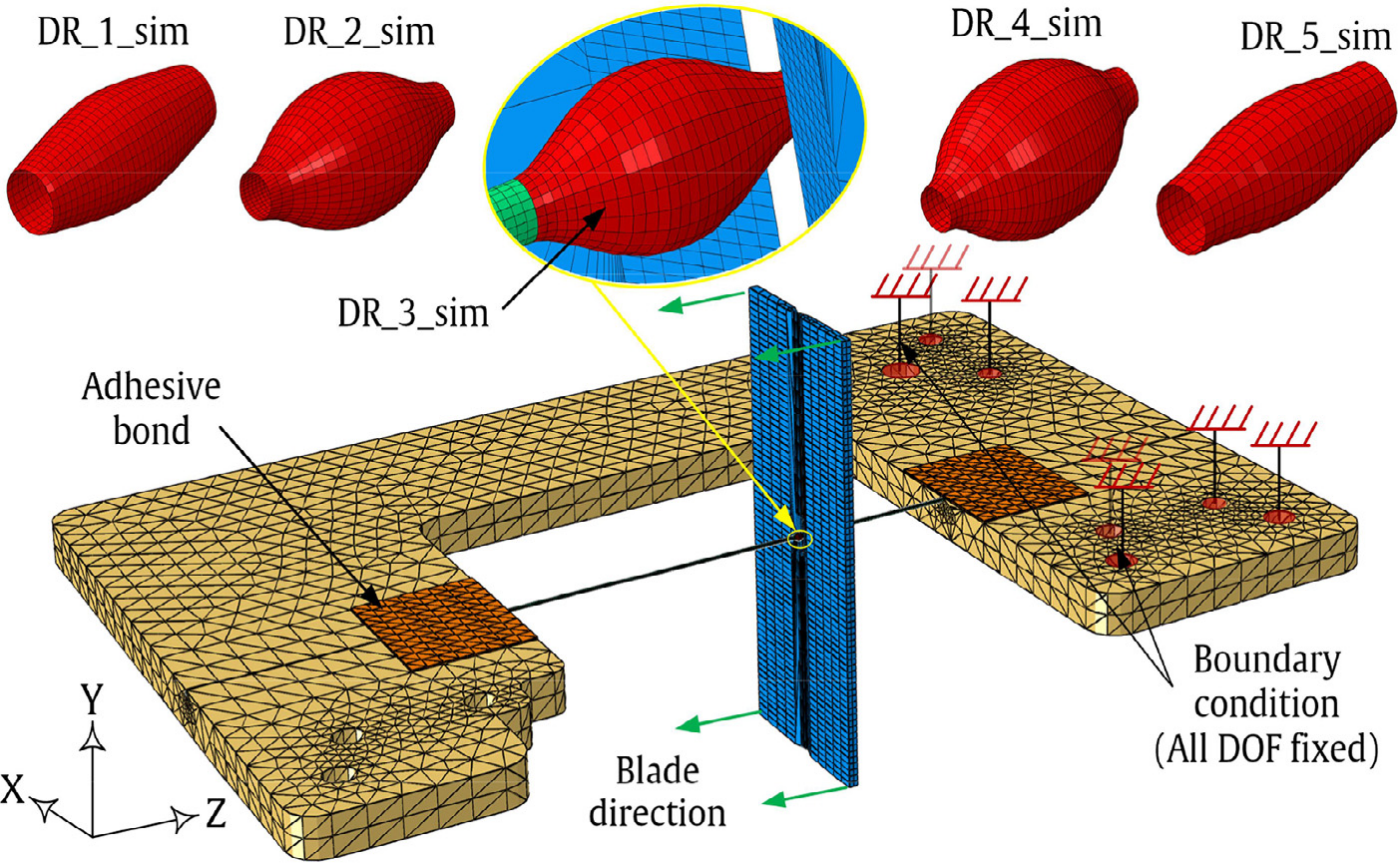
Finite element (FE) model of the MB test system and the different droplet models.

Corresponding numerical computation was conducted using a commercial ABAQUS Standard/2017 (Dassault Systèmes) [3] software code. The entire test was modelled in a 3D coordinate system, which includes the droplet, fibre, blades, connection by adhesive and the entire sample holder. As illustrated in Fig. 2, the fixed points of the sample holder were constrained in all degrees of freedom. The two ends of the fibres were constrained to the sample holder using modelled adhesive parts with a hard tie constraint. Material properties of the different constituents are described in Table 3. Cohesive Zone Modelling was deployed at the fibre matrix interface. The detailed configuration of the FEA and its models along with the interface model is described in the accompanying article [1].

The computations were run for five droplet configurations, different blade positions and the collected output data is given in this dataset. The strain data was extracted from a selected set of finite elements in the fibre model that undergoes tensile loading during the droplet loading simulation. The force data was extracted from the reference point in the rigid blade model. The input parameters for fitting $\varepsilon_{max}^{s}$ and $F_{max}^{s}$ are described in the accompanying article [1].

## Experimental force-strain data of droplets

**Fig. 3:**
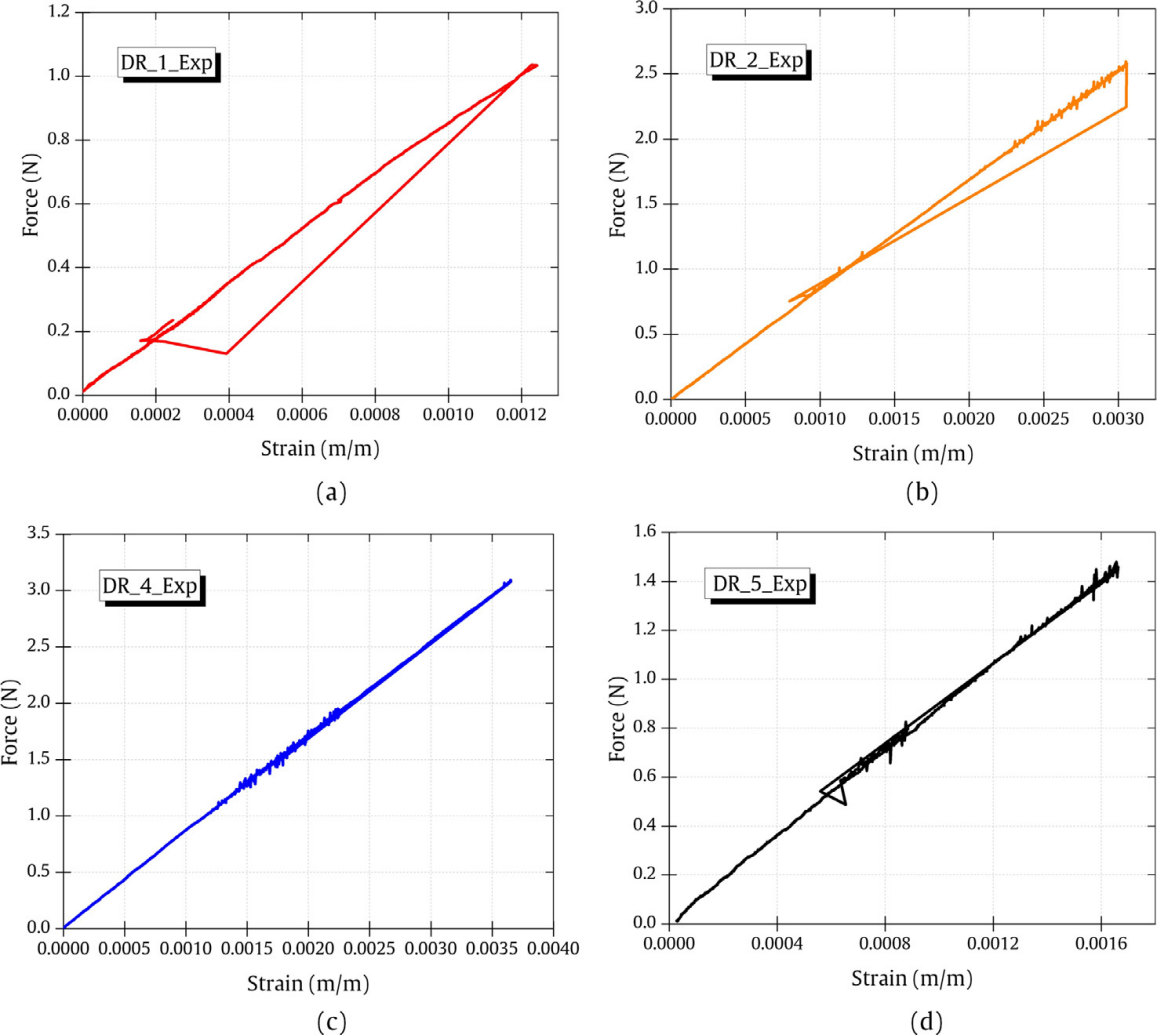
Force-strain data from experiments of (a) DR_1_Exp (b) DR_2_Exp (c) DR_4_Exp (d) DR_5_Exp. The complete number form of the data in raw and filtered format is included (experimental_data.xlsx).

The force data was recorded using FIBRObond microdroplet tester and the strain data was recorded using a Fiber Bragg Grating (FBG) interrogator at a sampling rate of 50 Hz. The recorded force-strain data of four different droplets (DR_i_Exp, i = droplet sample) is presented in Fig. 3.

## Debond load and Embedded area

**Fig. 4:**
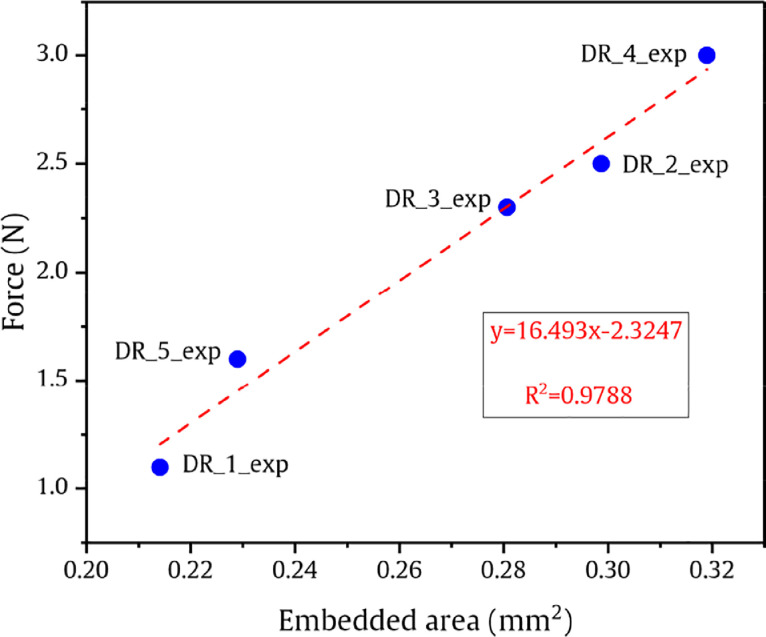
Debond load as a function of embedded area and a linear fit over the data points.

Fig. 4 shows the debond load as a function of embedded area for five different droplets used in the MB tests. The embedded area is given by the equation:

(1) $$A=\pi r^{2}l_{e}$$

wherein, *r* is the radius of the fibre (here 0.065 mm) and *l_e_* is the embedded length. The values of *l_e_* for five different droplets are tabulated in Table 2.

**Table 2:** Initial input parameters of this dataset based on the Shear lag equations.

Droplet No.	Droplet diameter (mm)	Embedded length *l_e_* (mm)	*F_d_* (N)	*V_1_*	*V_2_*	*G_c_* (J/m^2^)
DR_1_Exp	0.218	0.561	1.1	0.533415	0.466585	41.50
DR_2_Exp	0.384	0.731	2.6	0.171916	0.828084	49.40
DR_3_Exp	0.376	0.687	2.3	0.178903	0.821097	40.23
DR_4_Exp	0.452	0.786	3	0.124080	0.87592	48.65
DR_5_Exp	0.206	0.524	1.4	0.595946	0.404054	85.99

## Estimation of critical fracture energy using Shear lag model

Shear lag equations [4] were used to estimate the initial values of critical fracture energy (*G_c_*):

(2) $$G_{c}=\frac{rC_{33s}}{2}{\left[\frac{F_{d}}{\pi r^{2}}+\frac{D_{3s}\Delta T}{C_{33s}}\right]}^{2}$$

where *C*_33*s*_ and *D*_3*s*_ are the shear lag constants given by the below relations:

(3) $$C_{33s}=\frac{1}{2}\left[\frac{1}{E_{f}}+\frac{V_{1}}{V_{2}E_{m}}\right]\;\text{and}\;D_{3s}=\frac{1}{2}\left(\alpha_{f}-\alpha_{m}\right)$$

wherein *r* is the radius of the fibre (here 0.065 mm), *V_1_* and *V_2_* are the volume fraction of the fibre and droplet, respectively, Δ*T* is the temperature difference between the stress free temperature and the temperature of the droplet sample (here Δ*T* = 0°C), *E_f_* and *E_m_* are elastic modulus of fibre (here 70 GPa) and droplet (here 3.2 GPa), respectively, *F*_d_ is the debond force, *α_f_*  and *α_m_* are the co-efficients [1] of thermal expansion of fibre and droplet, respectively.

## Force and strain data from FE model simulation

**Fig. 5:**
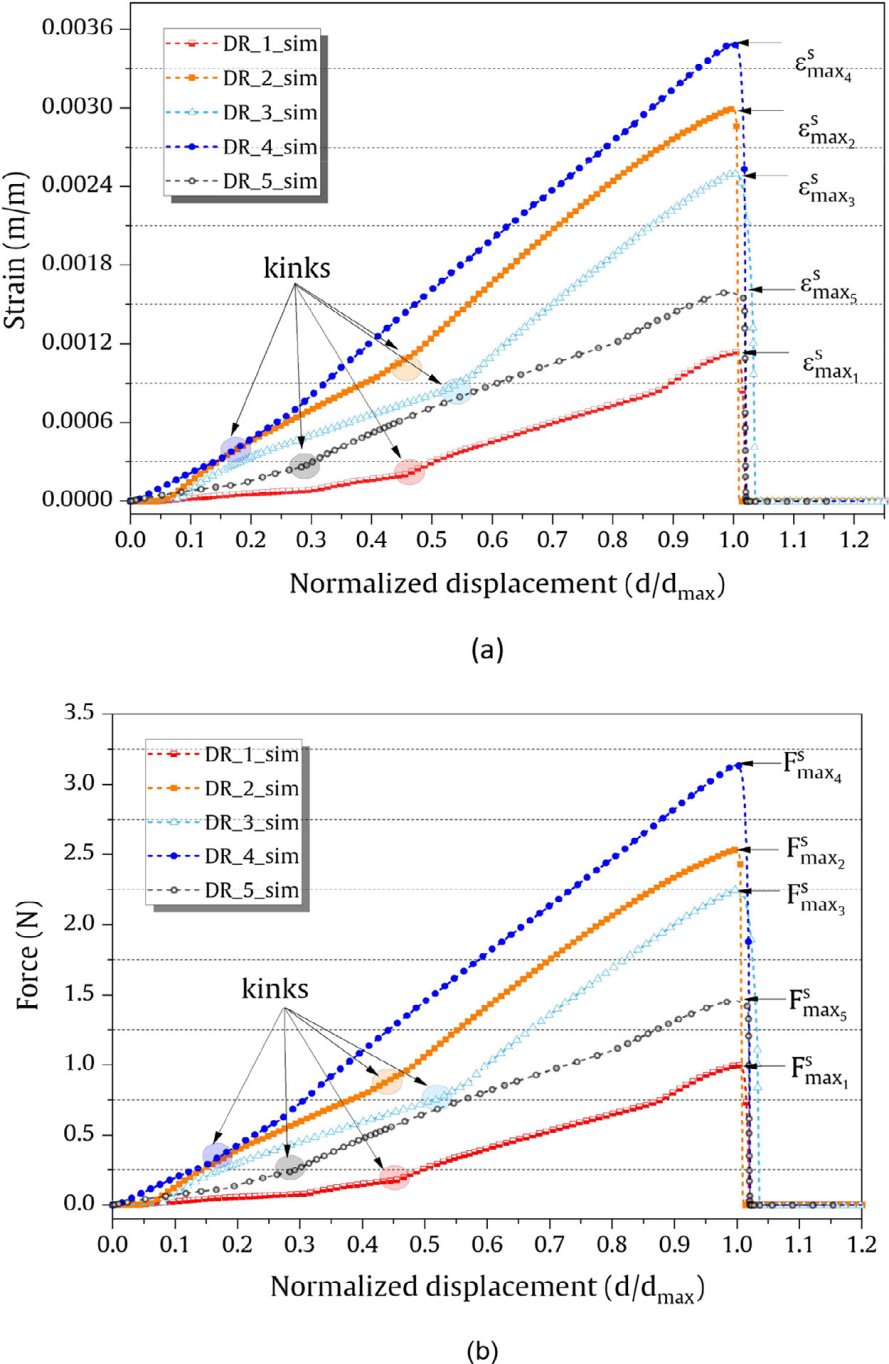
FE simulation data of the microbond tests: (a) strain-normalized displacement for the different droplets; (b) force-normalized displacement for the different droplets. The complete number form of the data of the graphs is included (force_strain_sim.xlsx).

**Fig. 6:**
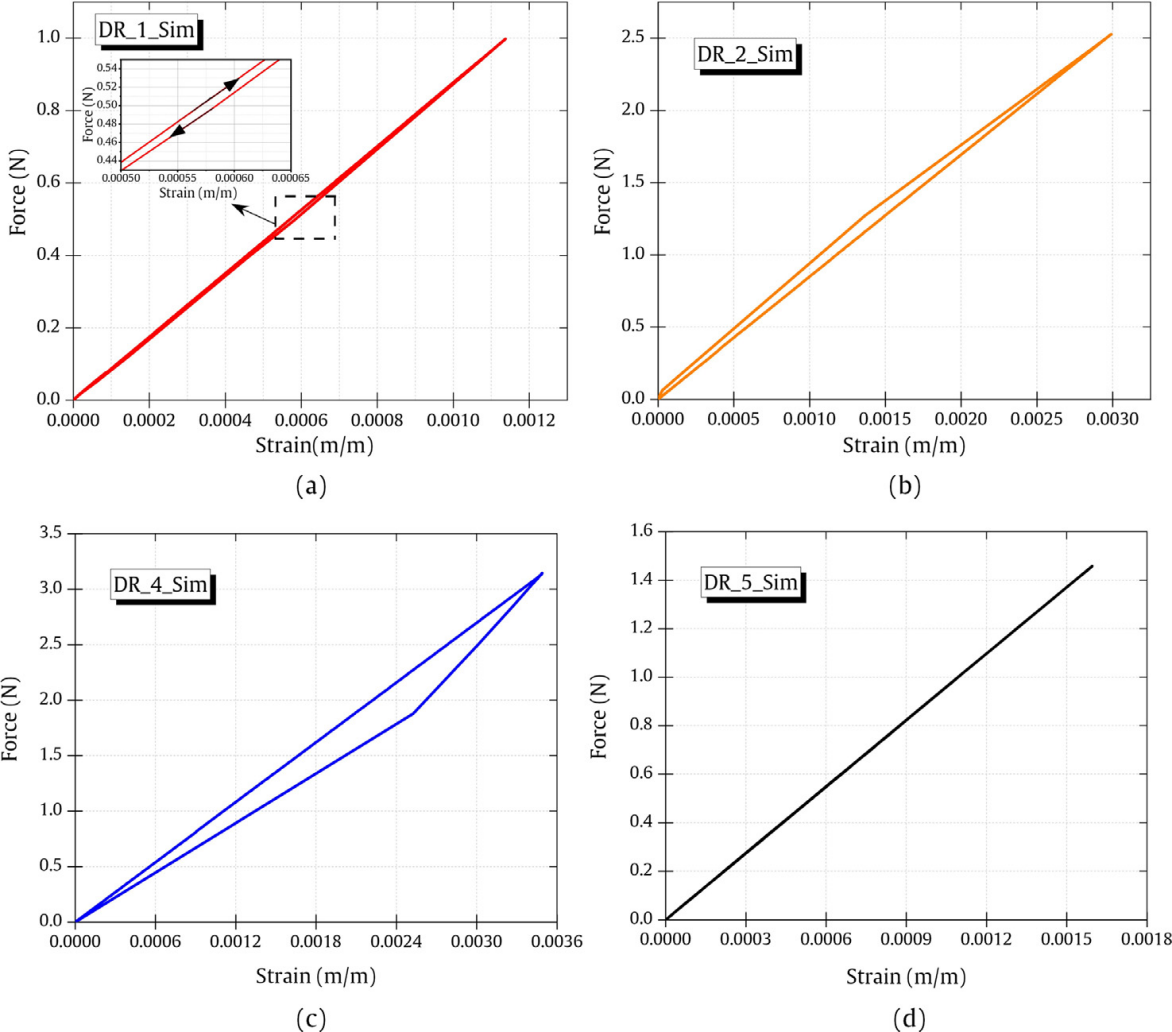
Force-strain data from FE simulations of different droplets (DR_i_Sim, i = droplet sample): (a) DR_1_Sim; (b) DR_2_Sim; (c) DR_4_Sim; (d) DR_5_Sim. The complete number form of the data of the graphs is included (simulation_data.xlsx).

The strain and force output data from the FE model simulation with the normalized displacement is shown in Fig. 5 (a) and Fig. 5 (b). Displacement was normalized with the maximum value of strain ($\varepsilon_{max}^{s}$) to be used in Fig. 5 (a) and with the maximum value of force ($F_{max}^{s}$) to be used in Fig. 5 (b) and for the different droplet sizes. The maximum value of strain and force after which debond occurred is indicated as $\varepsilon_{max_{1}}^{s}$- $\varepsilon_{max_{5}}^{s}$ and $F_{max_{1}}^{s}$- $F_{max_{5}}^{s}$ for the five droplets, respectively. The superscript ‘*s*’ stands for simulation, subscripts (1 to 5) stand for the different droplets. The simulated force-strain graphs for four different droplets are shown in Fig. 6. The graph inset in Fig. 6 (a) shows the direction (‘chronological’) of the loading curve and unloading curve after the droplet debonds.

## Blade position-related FE analysis data

**Fig. 7:**
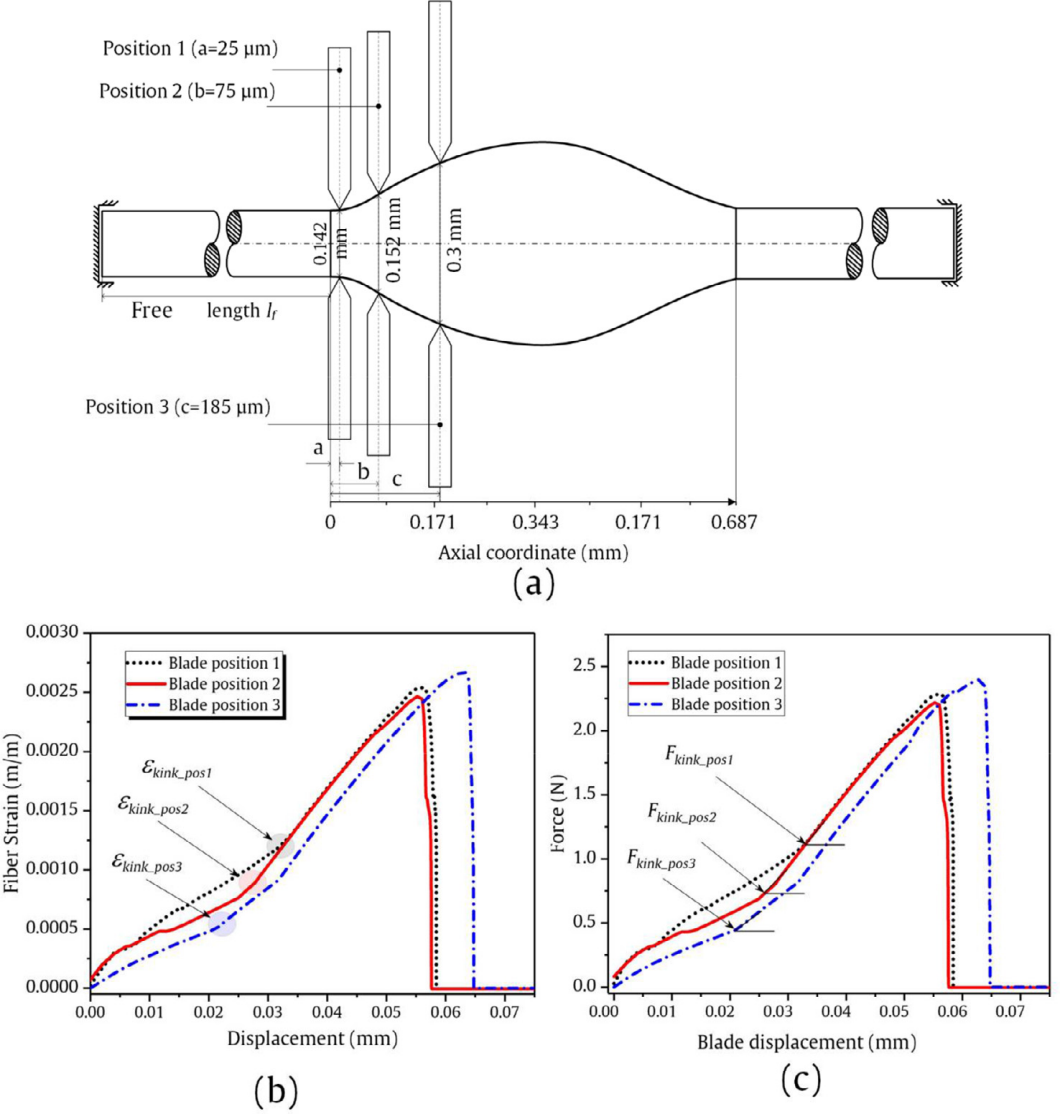
(a) Data corresponding to different blade positions and openings used in the FE model DR_3. (b) Data-related to the blade position in the strain-displacement output. (c) Data-related to the blade position in the force-displacement output. The complete output files are accompanied and available for readers (DR_3_position1.mp4, DR_3_position2.mp4, DR_3_position3.mp4).

The influence of blade position and blade opening during the FE simulation on the DR_3_Sim droplet model is demonstrated in Fig. 7. Three different blade opening distances and blade positions in contact with the droplet are shown in Fig. 7 (a). Position 1 has the least blade opening and Position 3 has the maximum blade opening distance. Corresponding strain and displacement (Fig. 7 (b)) and force-displacement graphs (Fig. 7 (c)) are here visualized and the data is available. The change in the blade position results in the shift of kink location whose detailed analysis is presented in a previous work [5]. As the FE simulation here is performed presuming quasi-static conditions, it was ensured that the blades were always in contact with the droplet model. The subscript ‘kink_pos1’ indicates the kink location at Position 1 (a = 25 µm).

## Mesh density-related data

**Fig. 8:**
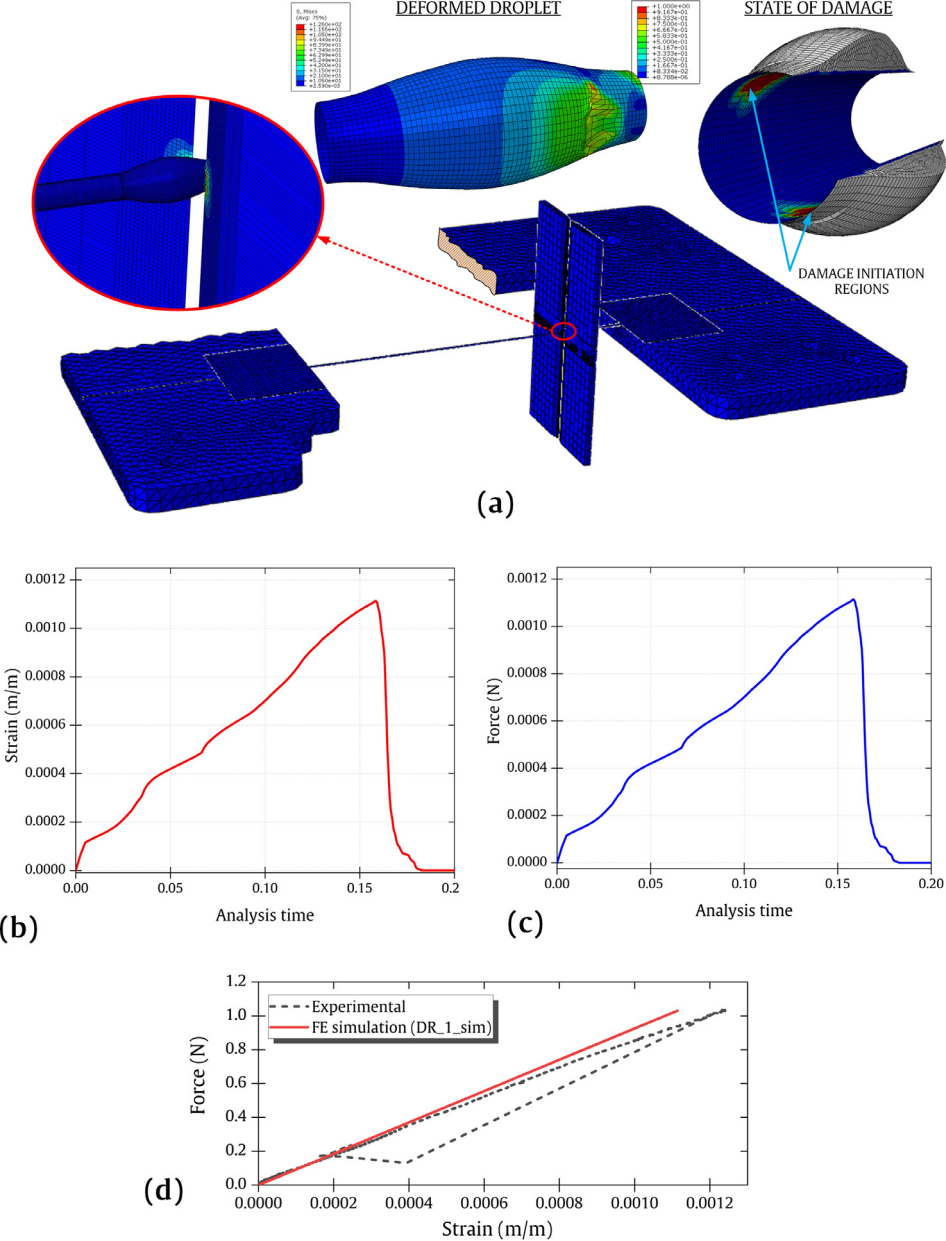
(a) FE simulation data of DR_1 with a fine mesh density along with the droplet deformation and damage initiation zone. (b) Strain-analysis time data for DR_1. (c) Force-analysis time data for DR_1. (d) Force as a function of strain as a combined data visualization of experimental and simulated data. The output and video data are accompanied in this work (fine_mesh.xlsx, microbond_1.mp4).

The FE modelling with a high-density element mesh was computationally solved and data visualization is presented in Fig. 8 (a). The model consisted of 309,628 elements that makes the model computationally expensive and it was solved using a supercluster [6]. Table 3 provides the details of the mesh density of the FE modelling here. The strain and force data as a function of analysis time are presented in Fig. 8 (b) and (c), respectively. Fig. 8 (d) shows the simulated and experimental force-strain data.

**Table 3:** The mechanical properties and FE mesh details used for the data collection in this dataset.

	Material properties		FE mesh details	
Constituent	Young's modulus (GPa)	Poisson's ratio	Element type	Total number of elements
FBG Fibre	70	0.22	C3D8R	197,627
Epoxy*	3.2	0.35	C3D8R	13,833
Blades	220	0.29	C3D8R	48,132
Sample holder	3.2	0.37	C3D4	47,956
Adhesive	1.6	0.29	C3D4	2520

C3D8R - 8 node linear brick reduced integration, C3D4R - 4 node linear tetrahedron reduced integration

⁎ Tri-linear plastic strain via kinematic hardening conditions with steps (0,0; 0,60; 0.002,70) [m/m, MPa] [7]

## Microscopy and FE simulation data of the deformed droplets

**Fig. 9:**
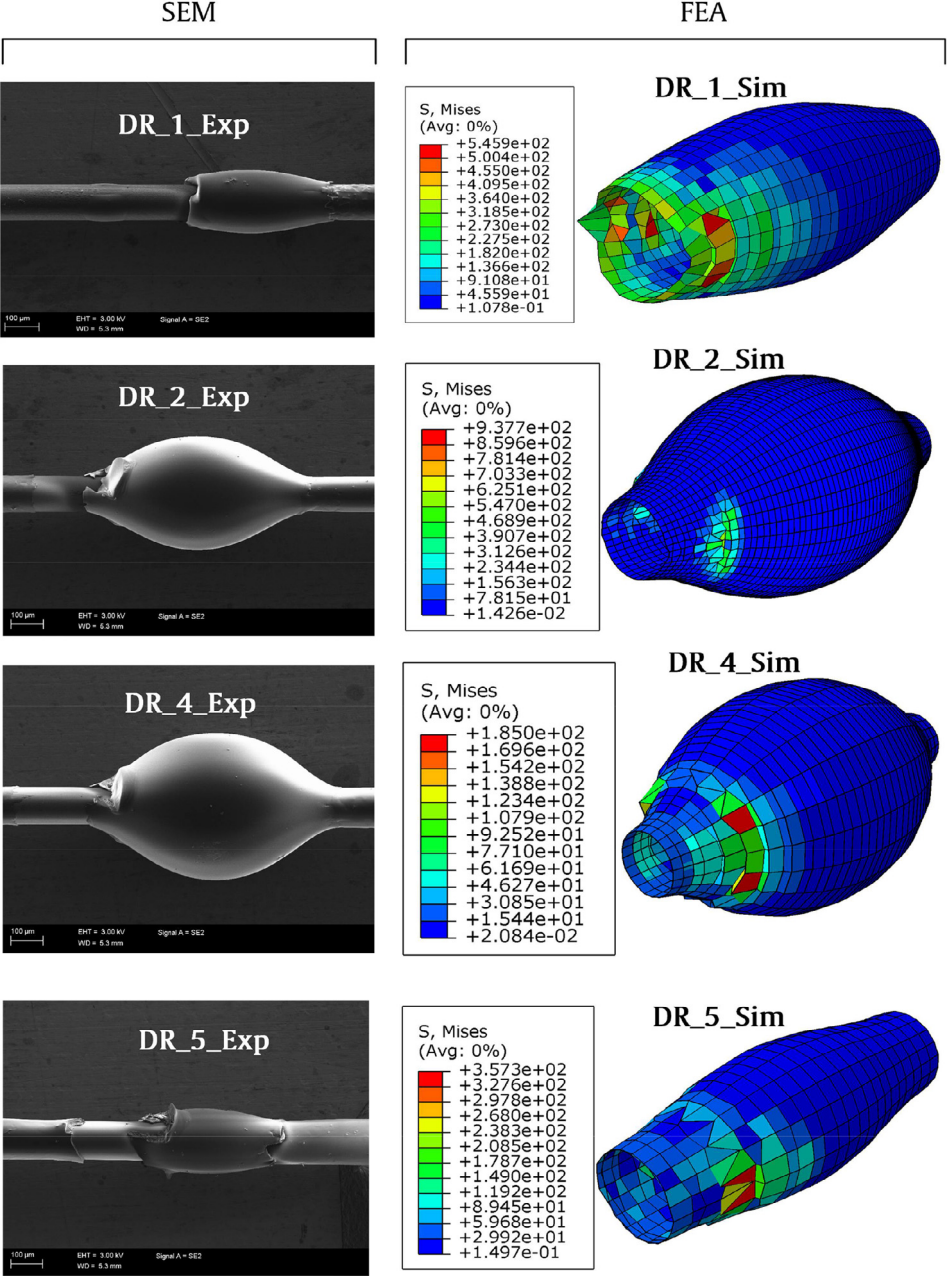
SEM imaging and FE simulation visualization (Von Mises stress included) of the droplets. High-resolution SEM imagings are accompanied with this work (SEM_1.tif, SEM_2.tif, SEM_4.tif, SEM_5.tif).

Corresponding deformed droplet visualizations from the FE computation (and their stress distribution visualization) and SEM generated imaging is presented in Fig. 9.

## Energy data of force derivatives in terms of strain

**Fig. 10:**
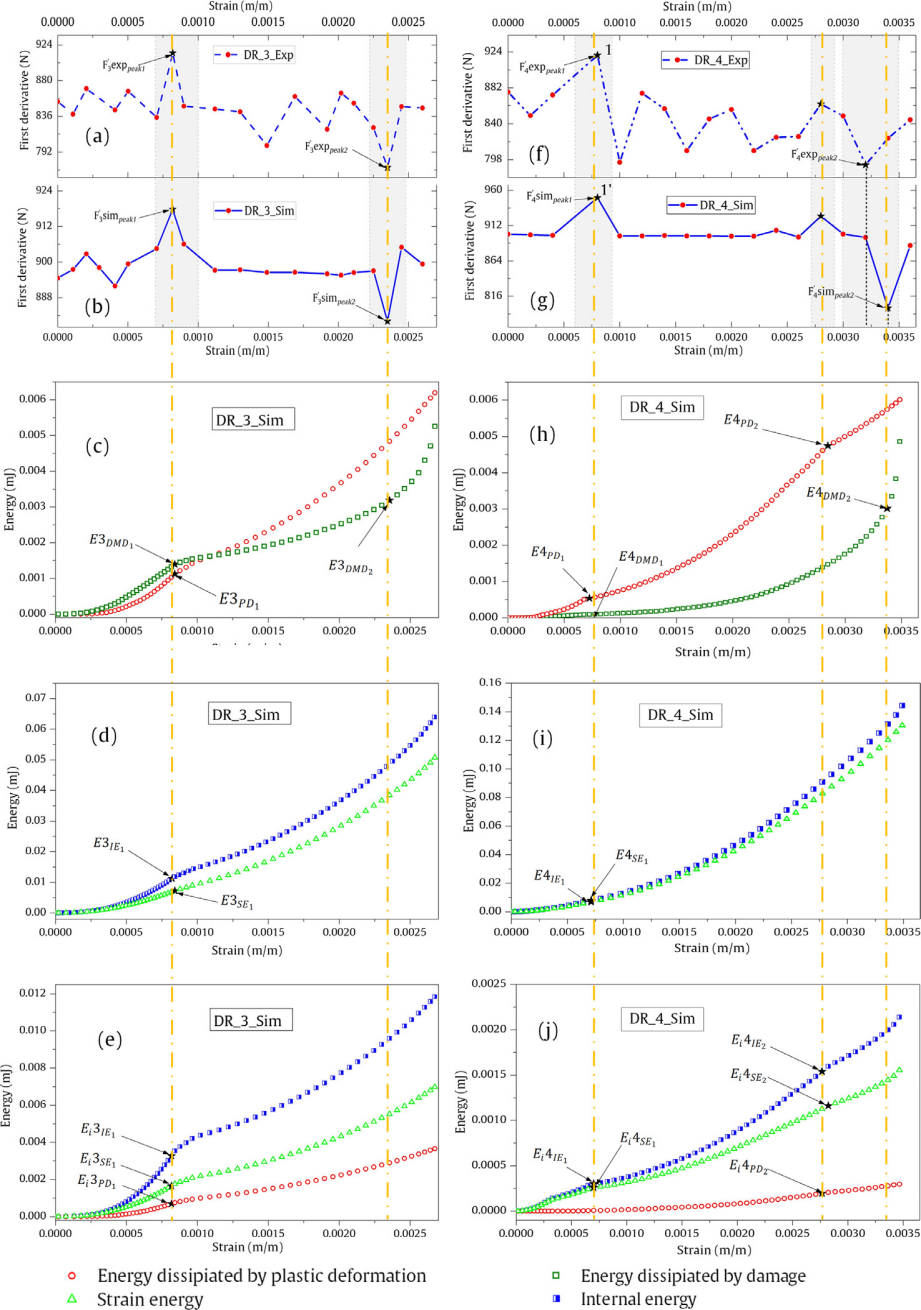
FE simulation output's visualization in terms of energy curves for DR_3 and DR_4.

Fig. 10 shows various energy output data to help reader understand the meaning of ‘peaks’ in the force-strain derivatives. DR_1 and DR_2-related distributions are presented in the previous work [1] whereas DR_3 and DR_4-related distributions are presented here. The data in Fig. 10 (a)–(e) represent DR_3-related simulation output and data in Fig. 10 (f)–(j) represent DR_4-related simulation output. Fig. 10 (c) shows the energy dissipated by plastic deformation (in the droplet model) and that by interfacial damage (via the cohesive zone interface). The internal and strain energy states of the entire model are visualized in Fig. 10 (d). Fig. 10 (e) shows the energy parameters, which are collected at the inner side of the droplet (model) along the interface (surface). This data helps in understanding the root cause for the peaks appearing in the first derivative data, which allows one to understand the fibre matrix interface in turn.

## Declaration of Competing Interest

The authors declare that they have no known competing financial interests or personal relationships which have, or could be perceived to have, influenced the work reported in this article.

## Appendix Supplementary materials

Image, application 1
